# Estimation of energy balance and training volume during Army Initial Entry Training

**DOI:** 10.1186/s12970-018-0262-7

**Published:** 2018-11-28

**Authors:** Jeremy McAdam, Kaitlin McGinnis, Rian Ory, Kaelin Young, Andrew D. Frugé, Michael Roberts, JoEllen Sefton

**Affiliations:** 1 Warrior Research Center, School of Kinesiology, Auburn University, 301 Wire Road, Auburn, AL 36849 USA; 2 Molecular and Applied Sciences Laboratory, School of Kinesiology, Auburn University, Auburn, AL 36849 USA; 3 Department of Cell Biology and Physiology, Edward Via College of Osteopathic Medicine (Auburn Campus), Auburn, AL 36849 USA; 4 Department of Nutrition, Dietetics, and Hospitality Management, College of Human Sciences, Auburn University, Auburn, AL 36849 USA

**Keywords:** Soldiers, Diet, Energy, Training, Volume

## Abstract

**Background:**

Adequate dietary intake is important for promoting adaptation and prevention of musculoskeletal injury in response to large volumes of physical training such as Army Initial Entry Training (IET). The purpose of this study was to evaluate training volume and dietary intake and estimate energy balance in Army IET soldiers.

**Methods:**

Dietary intake was assessed by collecting diet logs for three meals on each of three, non-consecutive days during the first week of IET. Training volume was measured across 13 weeks of training using Actigraph wGT3X accelerometers. Training intensity was classified using Sasaki vector magnitude three cut points. Energy expenditure estimates were calculated during weeks two and three of training using the modified Harris-Benedict equation and by estimation of active energy expenditure using metabolic equivalents for each classification of physical activity. All data is presented as mean ± standard deviation.

**Results:**

A total of 111 male soldiers (ht. = ± 173 ± 5.8 cm, age = 19 ± 2 years, mass = 71.6. ± 12.4 kg) completed diet logs and were monitored with Actigraphs. IET soldiers performed on average 273 ± 62 min low, 107 ± 42 min moderate, 26 ± 22 min vigorous, and 10 ± 21 min of very vigorous intensity physical activity daily across 13 weeks. The estimated total daily energy expenditure was on average 3238 ± 457 kcals/d during weeks two and three of IET. Compared to week one caloric intake, there was a caloric deficit of 595 ± 896 kcals/d on average during weeks two and three of IET. Regression analysis showed that body weight was a significant predictor for negative energy balance (adj. R2 = 0.54, *p* < 0.001), whereby a 1 kg increase in body mass was associated with a 53 kcal energy deficit.

**Conclusions:**

Based on week one dietary assessment, IET soldiers did not consume adequate calories and nutrients to meet training needs during red phase (weeks one through three). This may directly affect soldier performance and injury frequency. IET soldiers undergo rigorous training, and these data may help direct future guidelines for adequate nourishment to optimize soldier health and performance.

**Electronic supplementary material:**

The online version of this article (10.1186/s12970-018-0262-7) contains supplementary material, which is available to authorized users.

## Background

Initial Entry Training (IET) is a mentally and physically demanding military training program designed to transform civilians into soldiers. The transformation from civilian to soldier has become more challenging as the ability of IET soldiers to respond and adapt to IET has declined over recent decades. The American population has become less active due to changes in lifestyle factors such as increases in screen time in the place of physical activity, reductions in physically active jobs, and reduced active transportation (e.g., walking, riding bikes) [1]. Consequently, fitness levels of civilians entering IET are lower as evidenced by increases in failure rates on the initial fitness assessment of IET [2]. Lower physical fitness has been shown to be an important predictor of musculoskeletal injury (MSI) [2–6]. Soldier health and force readiness are greatly impacted by MSI, with MSI alone during IET estimated to cost the United States approximately $384 million per year [2, 7].

Soldiers perform organized physical fitness training as well as occupational physical activity to improve physical fitness and learn soldiering skills during IET [8–10]. Organized physical fitness training is designed to expose IET soldiers to progressively increasing levels of physical training to improve endurance and strength [9]. Occupational physical activity consists of tactical and survival drills that will enable IET soldiers to carry out required duties for the completion of successful missions. These physical and cognitive efforts must be adequately fueled in order to optimize IET soldier performance.

Rapid increases in training volume and intensity have been related to higher MSI rates [11]. Few investigations have quantified overall training volume including occupational physical activity. One study [12] quantified steps and distance covered by soldiers during IET and observed that those who walked more during basic combat training had a higher risk of musculoskeletal injury. To date, only one study [13] has investigated intensity of training in IET. However, the monitors were removed at dinnertime likely underestimating training volume.

Dietary intake must match training volume to fuel physiological demands. Inadequate energy is detrimental to bone health [14, 15], immune health [16, 17], cognitive performance [18, 19], as well as exercise performance in physically active populations [20, 21]. Research conducted on the dietary needs of active individuals has primarily focused on power or endurance training populations. IET soldiers are required to complete strength, power, endurance and functional training. This suggests IET soldiers have unique fueling needs due to the large variety in training intensity and duration. IET soldiers likely require the higher protein needs of strength and power athletes as well as the higher carbohydrate and fat intake needs of endurance athletes.

Clearly understanding training volume and nutrition intakes of IET soldiers is critical in ensuring training success. However, few studies to date have examined these areas in the IET environment, and none have examined both factors together in IET soldiers. Thus, the primary aims of this study were to: 1) quantify total training volume; 2) determine dietary intake; and 3) estimate energy balance in soldiers participating in IET. Secondary aims were to determine how dietary intake of IET soldiers compares to current nutritional recommendations for active individuals [22] and to determine if body size is a predictor of energy balance. We hypothesized IET soldiers would be in a negative energy balance and consume inadequate dietary intake. We also hypothesized that IET soldiers with heavier body mass would be at a greater risk for negative energy balance.

## Methods

### Ethical approval

The Auburn University Institutional Review Board, Army Institutional Review Board, and the Director, Research & Analysis Directorate Army Center approved the study procedures. For inclusion, participants had to be at least 18 years of age, healthy with no apparent disease or MSI, and participating in IET. Interested potential participants from one training unit received verbal explanation of the study from the study team and provided written consent. One hundred eleven male IET soldiers (mean ± SD: age: 19 ± 2 yrs., height: 173 ± 5.8 cm, mass: 72.2 ± 12.5 kg) from one training unit of IET soldiers at Fort Benning, Georgia volunteered for this investigation.

### Study design

This study was 14 weeks in total duration. Diet analysis and body mass information was collected during the first week of IET. Physical activity data was collected daily beginning at week two and continued ended during week 14 of IET training.

### Body mass measurement

Anthropometric measures were conducted in a fasted state prior to morning physical training and breakfast on the morning of day three of IET. Urine specific gravity was evaluated the morning of testing using a handheld refractometer (Manual, Atago, Tokyo, Japan) to ensure participants were properly hydrated (USG below 1.03) [23]. Height and weight were recorded with IET soldiers wearing only army issued physical training shorts, socks and underwear using a Health-O-Meter professional scale (Model 500KL, Sunbeam Products, Inc. Boca Raton, FL, USA).

### Diet logs

Dietary intake was recorded after each meal on three non-consecutive days (Tuesday, Thursday, and Saturday) during the first full week of IET. Food menus from the cafeteria-style dining facility serving the IET soldiers were used to create meal specific diet logs (Additional file 1) containing meal specific food items and serving sizes for each food item. Study staff provided guidance on identifying foods and quantifying portions prior to their first recorded meal. Items on the salad and fruit bars were measured in units relating to hand size whereas items explicitly listed on the dining facility menu were measured in standardized serving sizes (e.g., Lasagna-scoop). To assist participants a document was provided that contained written and visual representations of the relationships between food portion sizes relative to the hand. The IET soldiers were asked to circle the food item and portion they consumed. A member of the research team met the participants at the company barracks immediately following each meal to administer and obtain the diet logs.

### Diet log analysis

Macronutrient and select micronutrient data for the dining facility foods were accessed from the Army Joint Culinary Center of Excellence (JCOE) website [24]. Food items not found on the JCOE menu were retrieved from the US Department of Agriculture (USDA) nutrition data base [25]. Dietary intakes and nutrient information were entered into customized excel spreadsheets (Microsoft Excel, Microsoft Corporation, Redmond, WA, USA), and checked by two researchers to ensure data accuracy. Dietary intake calculations were completed using R statistical software [26] and R Studio [27]. R programming packages, dplyr [28], tidyr [29], reshape2 [30] ez [31], car [32], vars [33], ggplot2 [34]. Total calorie, protein, fat, carbohydrate, cholesterol, and sodium intakes were obtained for each meal and each day. Dietary intakes were then averaged across each day of diet logs to calculate daily averages. Participants who completed at least two full days of diet logs were used in calculation of average daily intakes and energy balance. However, for the statistical comparison of dietary intake at each meal, only participants who completed all three meals were used in the analysis. In total, 85 participants completed all 3 days of diet logs (included in the statistical analysis), and 26 participants completed only 2 days of diet logs.

### Physical activity assessment

Participants were outfitted with Actigraph wGT3X monitors (Actigraph, Pensacola, FL, USA). Training volume and intensity were measured across weeks 2–14 and energy balance was estimated for weeks two and three of red phase during IET. Monitors were initialized using Actilife software version 13.1.1 (Actigraph, Pensacola, FL, USA). Each week of training a member of the research team met with 12 soldiers (3 per platoon) to instruct them to wear the monitors around the waist, on the right side of the body, at all times (awake and asleep), and to only remove for showering. At the end of each week the monitors were collected, and age, height, weight, ethnicity, and hand dominance were entered into the Actilife software for each subject. Sasaki Vector Magnitude 3 cut points were used were used to divide activity into three categories: moderate (2690–6166 cpm), vigorous (6167–9642 cpm) and very vigorous (> 9642 cpm) [35]. A cut-point was added to classify counts less than moderate physical activity into sedentary and light intensity physical activity (Sedentary < 200 cpm, Light = 201–2689 cpm) which has been used in previous research as a cut point to delineate sedentary time from vector magnitude three data [36]. The monitors collected data at the recommended 30 Hz sampling rate [37]. Wear time validation was also used to estimate adherence to wearing the monitors. A day was considered valid if wear time was at least 600 min [36]. A total of 840 data points were eliminated from the analysis due to wear times less than 600 min. All training data was averaged per day and week of training. The data is presented in average minutes per day.

### Energy expenditure estimation

Energy expenditure was estimated during weeks two and three of IET. Minutes per day were averaged for each classification of physical activity and then metabolic equivalents (MET) were assigned to each category of physical activity. The MET assignments are as follows: light = 2 METs [38], Moderate = 3–5.99 METs, Vigorous = 6–8.99 METs, Very Vigorous > 9 METs (Sasaki) [35]. We conservatively applied the lowest MET values for moderate (3 METs), vigorous (6 METs), and very vigorous (9 METs) physical activity to estimate active energy expenditure [35]. Once MET values were assigned, average daily active energy expenditure (AEE) was estimated for each participant during weeks two and three using the Cooper Institutes MET to calorie conversion [39]:

$$ \mathrm{AEE}=\mathrm{MET}\ \mathrm{Value}\ \mathrm{x}\ \mathrm{Participant}\ \mathrm{body}\ \mathrm{weight}\ \left(\mathrm{kg}\right)\ \mathrm{x}\ \mathrm{Time}\ \left(\mathrm{hrs}.\right) $$

We estimated resting energy expenditure (REE) using the Modified Harris-Benedict equation [40]:

$$ \mathrm{REE}=88.362+\left(13.397\ \mathrm{x}\ \mathrm{body}\ \mathrm{weight}\ \left(\mathrm{kg}\right)\right)+\left(4.799\ \mathrm{x}\ \mathrm{Height}\ \left(\mathrm{cm}\right)\right)-\left(5.677\ \mathrm{x}\ \mathrm{age}\ \left(\mathrm{yrs}.\right)\right) $$

Total energy expenditure was the sum of REE and AEE. Energy balance was estimated by subtracting each participant’s caloric intake from the estimated total energy expenditure; negative or positive values indicate a caloric deficit or surplus, respectively.

### Data presentation and statistical analysis

Given that this study was largely observational, food intake, physical activity and energy balance data were presented as mean ± standard deviation values, and no statistical testing was performed for these metrics. Dietary intake across meals was evaluated using repeated measures ANOVA. Paired samples t-tests were used to evaluate the simple main effect of time and independent samples t-tests to evaluate the simple main effect of group when significant group by time interactions were found. Normality of residuals was tested using the Shapiro-Wilks and Komolgorov-Smirnov test. Sphericity was evaluated using Maulchy’s test. During tests for normality of residuals for dietary intake one meal was determined to be a statistical outlier and was removed from the analysis because the participant’s calorie intake was approximately 3500 cal for one meal, which was 2.5 times greater than his next closest meal (1427 cal). Linear regression was used to determine if body weight was a significant predictor of negative energy balance. Body weight was mean centered (participants body weight – mean (body weight)) was used as the predictor for average energy balance during weeks two and three of IET. For probability testing, statistical significance was set at *p* < 0.05.

## Results

### Physical activity data

**Fig. 1:**
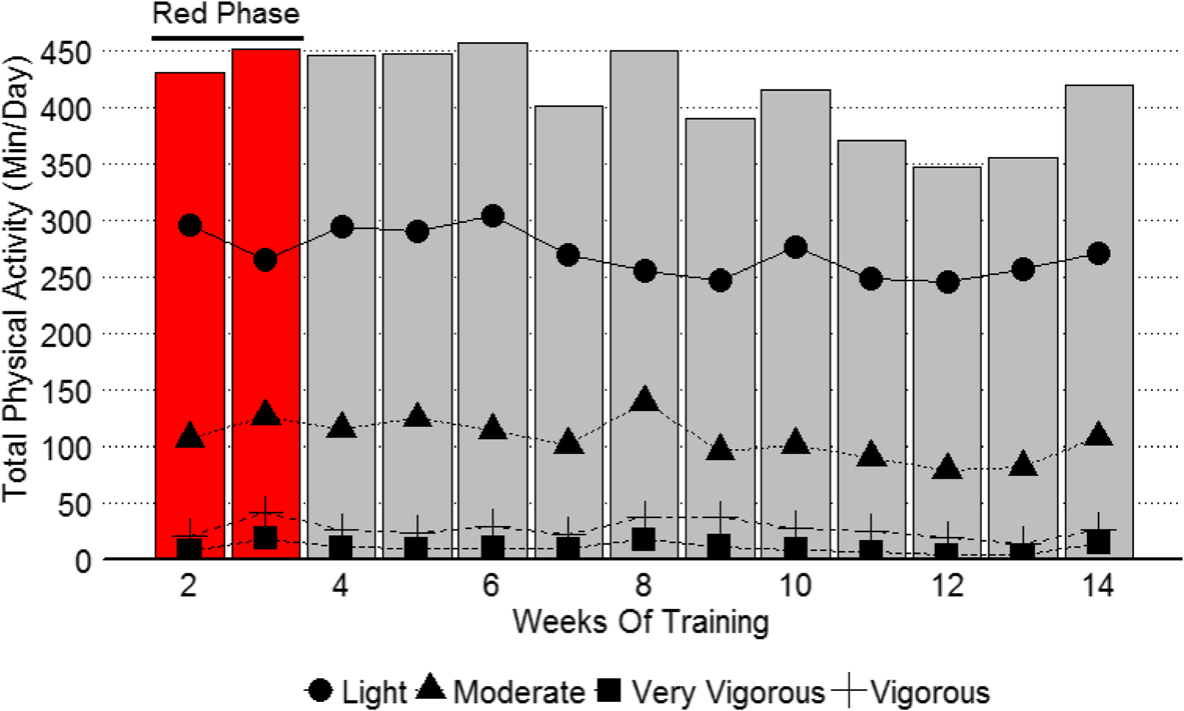
Summary of training volume across IET. Total physical activity (Sum of time spent in: light, moderate, vigorous, and very vigorous intensity) is represented by the columns and time spent in each classification of physical activity is represented by the lines. Data is presented in average minutes per day during each week of training

Physical activity data are presented in Fig. 1. On average IET soldiers spent approximately 273 ± 62 min in light, 107 ± 42 min in moderate, 26 ± 22 min in vigorous, and 10 ± 21 min in very vigorous intensity physical activity per day during weeks 2–14 of IET. Training volume during red phase was on average 284 ± 54 min in light, 114 ± 34 min in moderate, 28 ± 21 min in vigorous and 12 ± 17 min in very vigorous physical activity. Actilife software estimated the activity monitors were not worn (non-wear time) an average of 322 min per day. This non-wear time was inversely correlated with time spent in light intensity (Moderate Correlation: − 0.51) and moderate intensity (Small Correlation: − 0.33) exercise. Therefore, on weeks where wear time was lower, physical activity levels may be underestimated. IET soldiers averaged 13,569 ± 5197 steps per day during IET.

### Dietary intake

**Fig. 2:**
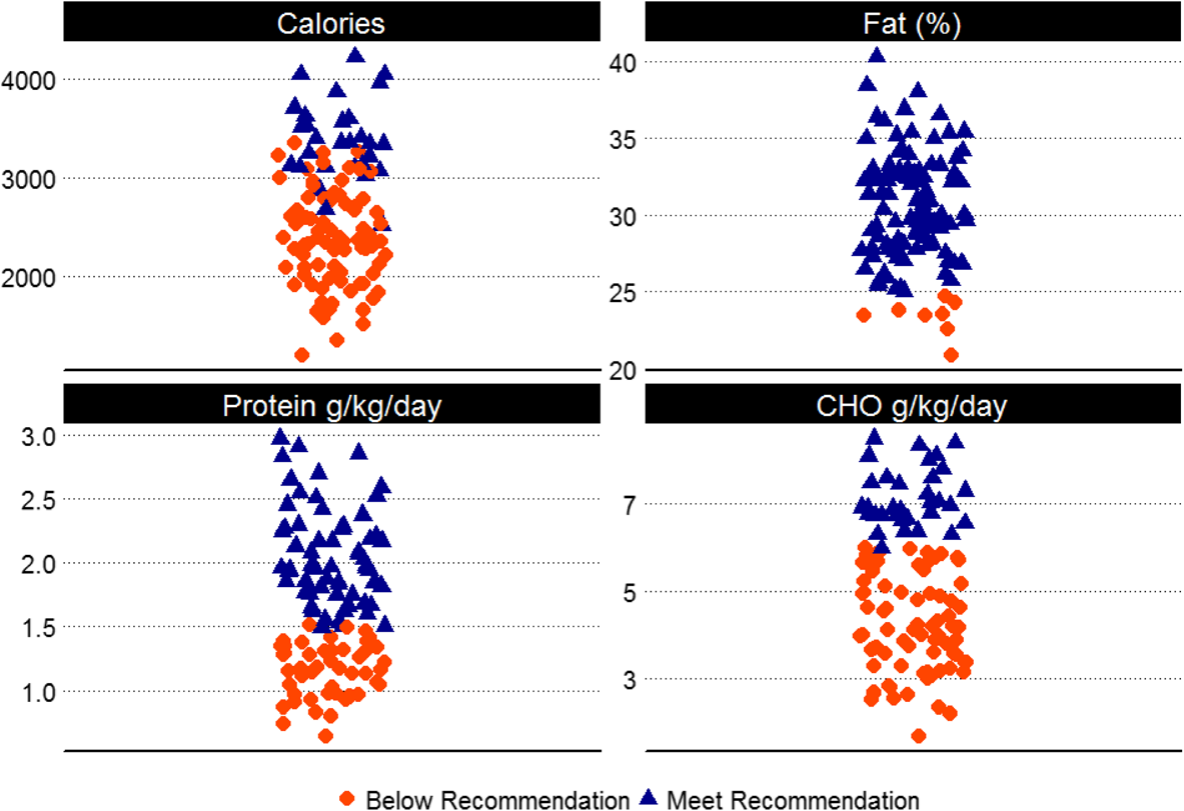
Summary of dietary intake in IET soldiers. Caloric intake (Calories per day), individuals who did not meet the recommendation were those who were in a negative energy balance; Protein, recommendation ≥1.5 g/kg body weight; Carbohydrate, recommendation ≥6 g/kg of body weight; Fat ≥25% total kcal intake, represent macronutrient intakes of IET soldiers in comparison to the recommended intake for active individuals [22]

Average macronutrient intake is presented in Fig. 2. IET soldiers consumed on average 2644 ± 639 cal per day during week one. Intake ranged from 1211 to 4228 cal per day. When comparing energy intake across meals, statistical comparison using repeated measures ANOVA revealed a significant main effect of meal with caloric intake highest at breakfast with an average intake of 1013 ± 273 cal, followed by dinner 813 ± 225 cal, and lunch 769 ± 201 cal (F = 69.1, *p* < 0.001). There was a significant difference between all meals for calorie intake, breakfast and dinner (*t* = 8.29, *p* < 0.001), breakfast and lunch (*t* = 10.72, p < 0.001), dinner and lunch (*t* = 2.31, *p* = 0.02). Protein intake averaged 38.1 ± 11.2 g for breakfast, 33.7 ± 9.9 g for lunch, and 41.1 ± 11.0 g for dinner. There was a significant effect of meal on dietary protein intake (F = 24.18, *p* < 0.001). Post-hoc paired t-tests revealed a significant difference between breakfast and dinner (*t* = 2.84, *p* = 0.005), breakfast and lunch (*t* = 3.95, *p* < 0.001), dinner and lunch (*t* = 7.14, p < 0.001). Carbohydrate intake averaged 137.3 ± 39.7 g for breakfast, 104.5 ± 28.2 g for lunch, and 101.8 ± 30.3 g for dinner. For carbohydrate intake, Greenhouse-Geisser corrections were used because sphericity was violated. Statistical analysis revealed a significant effect of meal. Post-hoc paired t-tests revealed a significant difference between breakfast and dinner (*t* = 9.03, *p* < 0.001), breakfast and lunch (*t* = 8.89, p < 0.001), but no significant difference between dinner and lunch (*t* = 0.95, *p* = 0.34). Fat intake averaged 34.6 ± 11.2 g for breakfast, 25.9 ± 9.2 for lunch, and 27.8 ± 11.2 g for dinner. Statistical analysis revealed a significant main effect of meal (F = 30.7, *p* < 0.001). Post-hoc paired t-tests revealed a significant difference between breakfast and dinner (*t* = 5.67, *p* < 0.001), breakfast and lunch (*t* = 7.67, *p* < 0.001), but no significant difference between dinner and lunch (*t* = 1.61, *p* = 0.11) for fat intake.

Approximately 49/111 participants consumed less than the recommended 1.5 g/kg of body weight per day of protein intake and 78/111 consumed less than the recommended 6 g/kg of carbohydrate per day. Approximately 103/111 met the lower limit of dietary fat intake (which is 25% of total calorie intake from fat).

### Energy balance

**Fig. 3:**
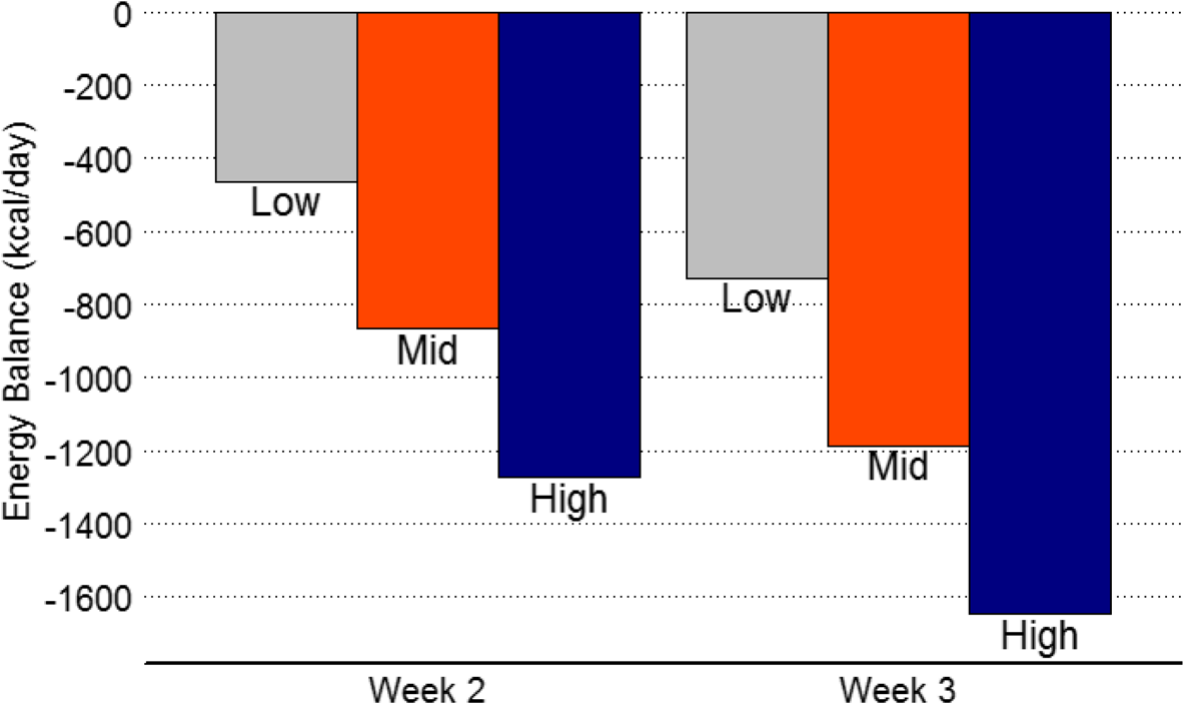
Energy balance estimate during red phase of IET. Energy balance is presented using three different MET values related to each classification of physical activity. Low, (Light = 2, Moderate = 3, Vigorous = 6). Mid, (Light = 2.5, Moderate = 4.5, Vigorous = 7.5); High, (Light = 2.99, Moderate = 5.99, Vigorous = 8.99) Very Vigorous was 9 METs across all estimates

Energy expenditure was estimated using the most conservative MET value associated with each classification of physical activity (Light = 2 METs, Moderate = 3 METs, Vigorous = 6 METs, Very Vigorous = 9 METs). Figure 3 summarizes energy balance during red phase of IET when the lowest MET values, average METs (Light = 2.5 METs, Moderate = 4.5 METs, Vigorous = 7.5 METs, Very Vigorous = 9 METs) and high MET (Light: 2.99 METs, Moderate = 5.99, Vigorous = 8.99, Very Vigorous = 9) values associated with each classification of physical activity. Average total energy expenditure was estimated to be 3238 ± 457 during red phase (week two: 3107 ± 415, week three: 3371 ± 461). Resting energy expenditure was estimated to be 1777 ± 185 and active energy expenditure was 1461 ± 286 (week two: 1329 ± 231, week three: 1593 ± 276) kcal kcal/day on average during red phase of IET. On average 71% of IET (week two: 66%, week three: 77%), soldiers were classified as being in negative net energy balance during red phase. Average energy balance was − 595 ± 896 kcal/day during red phase of IET based on week one dietary intake. Regression analysis revealed body weight was a significant predictor for negative energy balance (adj. R^2^ = 0.54, *p* < 0.001). Specifically, for every 1-kg increase in body mass, energy balance became more negative by 53 cal.

## Discussion

This study evaluated the dietary intake, training load, and balance between estimated energy expenditure and energy intake in U.S. Army IET soldiers. Our primary finding was that IET soldiers expended approximately 595 more calories per day during red phase than they consumed based on our estimation of metabolic training load. It is likely that our estimation of energy expenditure is low based on three important considerations: 1) we applied conservative MET assignments to moderate, vigorous, and very vigorous intensity physical activity; 2) estimation of BMR using the Modified Harris-Benedict equation was also a conservative estimate of non-physically active energy expenditure. It assumes the participant is immobile and in a supine position, whereas over half of sedentary time for IET soldiers is standing time which has been reported to increase energy expenditure by as much as 10% [41]; 3) IET soldiers frequently carry loads (10–80 pounds) which increases energy expenditure and was not considered in our calculations [13]. Therefore, the imbalance between energy expenditure and nutrition intake is likely higher than reported here. In addition to preventing optimal performance and recovery, inadequate energy intake can negatively affect bone turnover (formation and breakdown), and may predispose active individuals to musculoskeletal injury [14, 15, 42, 43] which is one of the most costly challenges facing the US armed forces today [2, 7].

Quantification of training volume during IET revealed soldiers averaged approximately 3180 ± 320 min of physical activity per week with 1202 ± 291 of those minutes being moderate to vigorous physical activity across 14 weeks of IET. For comparison, research indicates only 49–53% of civilian adults 18–34 reported at least 150 min of moderate or 75 min of vigorous and only 31% reported participating in 300 min of moderate or 150 min of vigorous physical activity per week [44]. Furthermore, only 27% of adolescents participate in more than 1 h per day of moderate or vigorous physical activity [9]. Thus, IET soldiers complete substantially more physical activity than the general civilian population of the same age range. Also noteworthy was our observation that physical activity was higher during the first three weeks compared to the overall cycle average for IET training. Thus, IET soldiers may experience rapid increases in physical activity upon entry to IET compared to civilian life, which has been reported to increase the risk of MSI [11]. To combat this disparity, there are several voluntary pre-conditioning programs provided to new U. S. Army recruits. Discussions with training and recruiting command Cadre suggest most recruits do not take advantage of these programs or guidance provided on how to prepare for the rigors of military training. A different approach for pre-conditioning of recruits may be necessary to prepare them for the rigors of IET.

Previous research reported that IET soldiers spent on average 140 min per day in light, approximately 92 min in moderate, and 38 min per day in vigorous intensity exercise (averages calculated from cumulative results from Fort Sill and Fort Jackson) [13]. Differences found in the current study are likely due methodological differences in activity monitoring. The previous study distributed activity monitors at breakfast and collected them at dinner, missing any evening physical activity [13]. Additionally, differences in algorithms used to classify activity counts into physical activity may account for the differences. The prior study used Freedson cut points which are based on vertical axis counts [36, 45], whereas our study used Sasaki vector magnitude cut-points which are based on a vector magnitude estimate calculated from all three axes [35]. While vertical axis counts have been validated for recording ambulatory physical activity (walking and jogging), they tend to underestimate activities that do not necessarily occur in the vertical axis (such as shoveling) [46, 47]. Comparison of models using vector magnitude (tri-axis) and vertical axis only have reported higher physical activities when using vector magnitude whereas vertical axis tends to report larger amounts of time in sedentary activity [46, 48].

Soldiers consumed an average of 2644 cal, 114 g of protein, 352 g of carbohydrate, and 89 g of fat per day. Our results are limited as we only report week one dietary intake. A 2002 study of dietary intakes in IET soldiers found they consumed on average 3000 cal per day, whereas a 2012 study found IET soldiers consumed on average 1975 cal (78 g of protein, 240 g of carbohydrate, and 77 g of fat) [49, 50]. Differences in dietary intake results may also be due to methodologies employed in collecting nutritional data. The first study conducted in 2002 used a highly accurate food photography method whereas the study conducted in 2012 used food frequency questionnaires and the current investigation utilized diet logs. Additionally, it should be noted that the previous studies used serial measures (pre and post) for dietary assessment, whereas we only report week one dietary intake. Even though differences exist, our results for calorie intake still fall within the reported calorie intakes of these two studies. Previous research has reported factors such as not having enough time to eat and command climate can influence nutritional behaviors [51]. Special care to protect feeding times needs to be considered especially when the influx of IET soldiers increases due to force expansion or yearly fluctuations, which may further reduce the time allotment for meals due to increased demand on dining facilities. Additionally, many doctrine changes to the IET environment have been made during this period. Early in 2012 and 2013 the U.S. Army began using strategies for food education and selection to encourage IET soldiers to avoid consuming energy dense food items. Soldiers were encouraged to select foods lower in fat content and increase consumption of vegetables, fruits and complex carbohydrates [52, 53]. While this nutritional education is important for development of long-term soldier health, care should be taken when explaining these strategies to IET soldiers who are exposed to very high training volumes and need energy dense food items to match caloric expenditure.

Protein intake is crucial for provision of amino acid pools to support cellular adaptations of skeletal muscle such as hypertrophy [54], mitochondrial protein turnover [55, 56], repair of muscle damage caused by training [57, 58], bone health [59–61] and improved performance [22]. On average IET soldiers consumed more than the lower limit of 1.2 g/kg for protein. However, approximately 44% of IET soldiers consumed less than the recommended 1.5 g/kg of protein intake per day, which is thought to be a more accurate estimation of protein needs for strength and endurance training [22, 62, 63]. Additionally, protein needs for active individuals may increase to 2–3 g/kg of body weight per day when training during energy restriction [64]. This suggests that even though IET soldiers consumed above the lower limit for daily protein intake, they are likely consuming inadequate amounts of protein in due to the training demands and energy restriction soldiers face during IET.

Diets high in carbohydrate intake are associated with increased power output [65, 66], prolonged time to exhaustion [67], and overall increases in exercise performance [66]. We report that approximately 50% of IET soldiers did not consume more than 5 g/kg and 70% did not meet the lower limit (6 g/kg) of the recommendations for carbohydrate for individuals participating in high levels of physical activity [22]. Conversely, 93% of IET soldiers met the lower recommendation of 25% of total calorie intake from fat. These findings suggest that carbohydrate intake may be inadequate to meet the demands of training and thus may limit performance in IET soldiers. This is important as we apply research findings clinically to develop fueling recommendations for the IET population. Currently all IET soldiers receive the same serving size and amount at the dining facility. Individual soldier body composition, fitness and activity level should be considered as we work towards optimization of health and performance.

This investigation is a first step at evaluating energy balance in the IET environment and represents only one training company at one location. One limitation to this study is our assumption that entry-level dietary intake values were reflective of eating habits throughout the 14-week duration. Additionally, we did not estimate energy expenditure during the same week in which dietary data was collected. However, training schedule and amount of time to eat are similar within red phase (weeks 1–3) and therefore dietary intake was likely similar in weeks two and three compared to what was found in week one. Additionally, energy expenditure was estimated based on the average physical activity performed by the entire company of IET soldiers. While many of the activities performed at IET are in the group setting, ideally, investigations should assign each IET soldier a monitor to wear daily throughout training to track individual energy expenditure and training load. Finally, using serial and more precise methods to track dietary intake would improve outcomes. There are known inherent errors when using food logs such as portion estimation error, therefore methodologies in which trained researchers evaluate dietary intake may improve accuracy [68].

## Conclusion

Our results suggest that based on the week one diet assessment, the IET soldiers in this study did not consume adequate nutrition to meet the physiological demands of training. This may impair each soldiers training response and potentially increase injury rates associated with recovery and fatigue. Additional research is needed to develop strategies to optimize soldier nutrition to improve performance and recovery.

## Additional file

Additional file 1: **Table S1.** Example food log. (DOCX 20 kb)

## Abbreviations

AEE: Active Energy Expenditure
IET: Initial Entry Training
JCOE: Army Joint Culinary Center of Excellence
MET: Metabolic Equivalent
MSI: Musculoskeletal Injury
REE: Resting Energy Expenditure
USDA: United States Department of Agriculture
