# Supplementary material for: Estimation of energy balance and training volume during Army Initial Entry Training
Source: J Int Soc Sports Nutr. 2018 Nov 28;15:55. doi: 10.1186/s12970-018-0262-7 (PMC6264031; doi:10.1186/s12970-018-0262-7)
Supplement: Supplementary file 1 — Table S1. Example food log. (DOCX 20 kb) [file 12970_2018_262_MOESM1_ESM.docx]

Table S1. Example food log

| **Roster Number: _________** |  | **Dinner** | |  |  |  |  |  |  |  |  |  |
| --- | --- | --- | --- | --- | --- | --- | --- | --- | --- | --- | --- | --- |
| **Please circle the food you ate** |  | **Please Circle the portion you ate** | | | | | | | | | | |
| Lasagna |  | 1/2 | scoop |  | 1 | scoop |  | 1.5 | scoop |  | _____ | scoop |
| Chicken |  | 1/2 | piece |  | 1 | piece |  | 1.5 | piece |  | _____ | piece |
| Vegetarian Cheese Manicotti |  | 1/2 | scoop |  | 1 | scoop |  | 1.5 | scoop |  | _____ | scoop |
| Orzo-Spinach, tomato, onion |  | 1/2 | scoop |  | 1 | scoop |  | 1.5 | scoop |  | _____ | scoop |
| Mashed potatoes |  | 1/2 | scoop |  | 1 | scoop |  | 1.5 | scoop |  | _____ | scoop |
| Green bean combo |  | 1/2 | scoop |  | 1 | scoop |  | 1.5 | scoop |  | _____ | scoop |
| Squash |  | 1/2 | scoop |  | 1 | scoop |  | 1.5 | scoop |  | _____ | scoop |
| Gravy (chicken) |  | 1/2 | scoop |  | 1 | scoop |  | 1.5 | scoop |  | _____ | scoop |
| Rolls |  | 1/2 | roll |  | 1 | roll |  | 1.5 | roll |  | _____ | roll |
| Soup |  | 1/2 | scoop |  | 1 | scoop |  | 1.5 | scoop |  | _____ | scoop |
| **Salad bar** |  |  |  |  |  |  |  |  |  |  |  |  |
| Salad |  | 1 | fist |  | 2 | fist |  | 3 | fist |  | _____ | fist |
| Tomatoes |  | 2 | tomatoes |  | 3 | tomatoes |  | 4 | tomatoes |  | _____ | tomatoes |
| Peppers |  | 1/2 | handful |  | 1 | handful |  | 1.5 | handful |  | _____ | handful |
| Cheese |  | 1/2 | handful |  | 1 | handful |  | 1.5 | handful |  | _____ | handful |
| Other _____________ |  |  |  |  |  |  |  |  |  |  |  |  |
| Other _____________ |  |  |  |  |  |  |  |  |  |  |  |  |
| Other _____________ |  |  |  |  |  |  |  |  |  |  |  |  |
| Salad dressing ____________ |  | 1 | thumb |  | 2 | thumb |  | 3 | thumb |  | _____ | thumb |
| **Fitness bar** |  |  |  |  |  |  |  |  |  |  |  |  |
| Fruit _________________ type | | 1/2 | handful |  | 1 | handful |  | 1.5 | handful |  | _____ | handful |
| Fruit _________________ type | | 1/2 | fruit |  | 1 | fruit |  | 1.5 | fruit |  | _____ | fruit |
| Fruit _________________ type | | 1/2 | handful |  | 1 | handful |  | 1.5 | handful |  | _____ | handful |
| Fruit _________________ type | | 1/2 | fruit |  | 1 | fruit |  | 1.5 | fruit |  | _____ | fruit |
| Yogurt _________________ type |  | 1/2 | cup |  | 1 | cup |  | 1.5 | cup |  | _____ | cup |
| Other _____________ |  |  |  |  |  |  |  |  |  |  |  |  |
| **Drinks** |  |  |  |  |  |  |  |  |  |  |  |  |
| Milk |  |  |  |  |  |  |  |  |  |  |  |  |
| Water |  |  |  |  |  |  |  |  |  |  |  |  |
| Juice _________________ type |  | 1/2 | cup |  | 1 | cup |  | 1.5 | cup |  | _____ | cup |
| **Other** |  | 1/2 | cup |  | 1 | cup |  | 1.5 | cup |  | _____ | cup |
| Butter | | 1/2 | cup |  | 1 | cup |  | 1.5 | cup |  | _____ | cup |
| Honey |  |  |  |  |  |  |  |  |  |  |  |  |
| Peanut butter |  | 1 | thumb |  | 2 | thumb |  | 3 | thumb |  | _____ | thumb |
| Jelly |  | 1 | thumb |  | 2 | thumb |  | 3 | thumb |  | _____ | thumb |
| Relish |  | 1 | thumb |  | 2 | thumb |  | 3 | thumb |  | _____ | thumb |
| A1 |  | 1 | pack |  | 2 | pack |  | 3 | pack |  | _____ | pack |
| Hot sauce |  | 1 | thumb |  | 2 | thumb |  | 3 | thumb |  | _____ | thumb |
| Heinz 57 sauce |  | 1 | thumb |  | 2 | thumb |  | 3 | thumb |  | _____ | thumb |
| Ketchup |  | 1 | thumb |  | 2 | thumb |  | 3 | thumb |  | _____ | thumb |
